# Supplementary material for: Doping-control of excitons and magnetism in few-layer CrSBr
Source: Nat Commun. 2024 Jun 3;15:4735. doi: 10.1038/s41467-024-49048-9 (PMC11535057; doi:10.1038/s41467-024-49048-9)
Supplement: Supplementary file 1 — Supplementary Information [file 41467_2024_49048_MOESM1_ESM.pdf]

SUPPLEMENTARY INFORMATION: Doping-control of excitons and magnetism in few-layer CrSBr

Farsane Tabataba-Vakili,[1,2] Huy P. G. Nguyen,[1] Anna Rupp,[1] Kseniia Mosina,[3] Anastasios Papavasileiou,[3] Kenji Watanabe,[4] Takashi Taniguchi,[5] Patrick Maletinsky,[6] Mikhail M. Glazov,[7] Zdenek Sofer,[3] Anvar S. Baimuratov,[1] and Alexander Högele[1,2]

[1]*Fakultät für Physik, Munich Quantum Center, and Center for NanoScience (CeNS), Ludwig-Maximilians-Universität München, Geschwister-Scholl-Platz 1, 80539 München, Germany*

[2]*Munich Center for Quantum Science and Technology (MCQST), Schellingtraße 4, 80799 München, Germany*

[3]*Department of Inorganic Chemistry, University of Chemistry and Technology Prague, Technická 5, 166 28 Prague 6, Czech Republic*

[4]*Research Center for Functional Materials, National Institute for Materials Science, 1-1 Namiki, Tsukuba 305-0044, Japan*

[5]*International Center for Materials Nanoarchitectonics, National Institute for Materials Science, 1-1 Namiki, Tsukuba 305-0044, Japan*

[6]*Department of Physics, University of Basel, Basel, Switzerland*

[7]*Ioffe Institute, 194021 Saint Petersburg, Russian Federation*

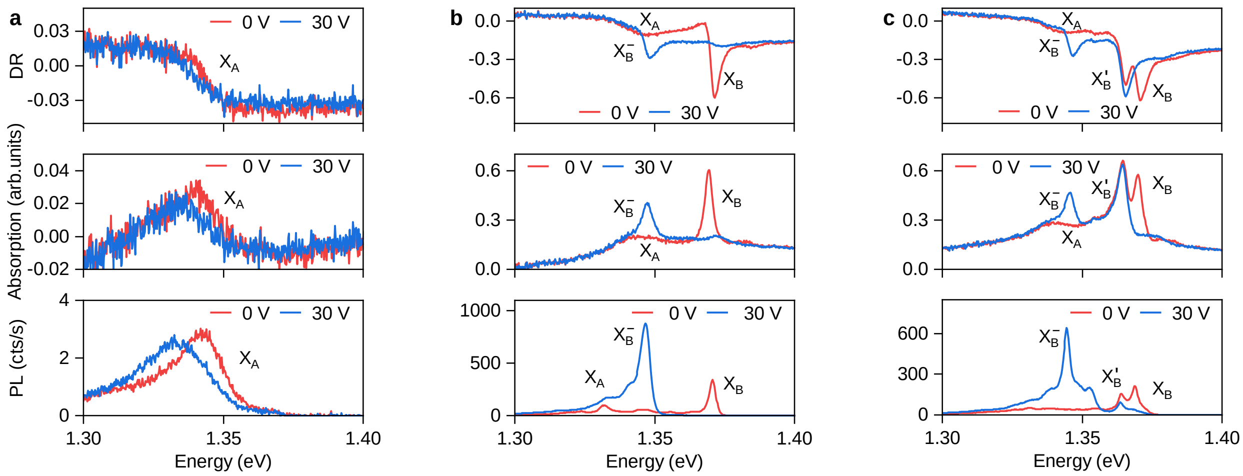

Supplementary Fig. 1. **Differential reflectance, absorption, and photoluminescence spectra.** **a**, Monolayer DR (top), absorption (middle), and PL spectra (bottom) at 0 and 30 V. **b**, and **c**, Same as **a** but for the bilayer and trilayer, respectively. X_A, X_B, and X_B' label neutral and X_B^- charged exciton transitions. DR: differential reflectance, PL: photoluminescence.

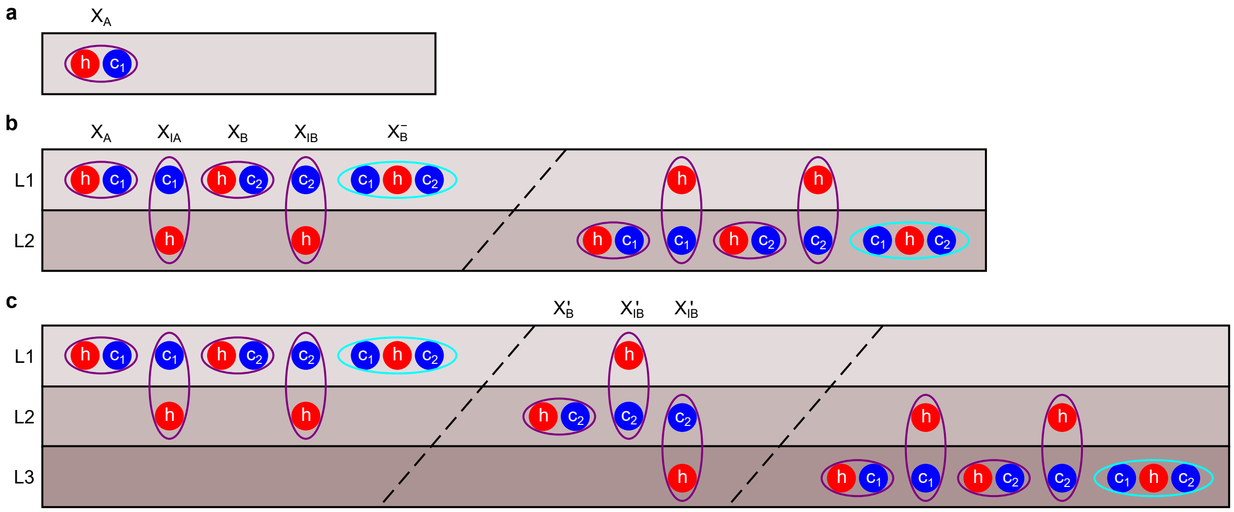

Supplementary Fig. 2. **Neutral and charged excitons considered in mono-, bi- and trilayer.** **a** – **c**, Intra- and interlayer excitons and intralayer, interband charge-bound states in mono- (**a**), bi- (**b**), and trilayer (**c**). The dashed lines in **b** and **c** separate the different blocks in the interaction Hamiltonians in Eqs. (1), (2), (4), and (5) in Methods for electrons in layers L1, L2, and L3, respectively. Electrons (holes) are shown in blue (red), c_1 and c_2 refer to the electron conduction subbands. X_A (X_B) are intralayer excitons with the electron in the c_1 (c_2) subband, and X_{IA} and X_{IB} are respective interlayer excitons. Prime ($'$) denotes states of L2 in the trilayer with a different dielectric environment and without bound states with electrons in the c_1 band.

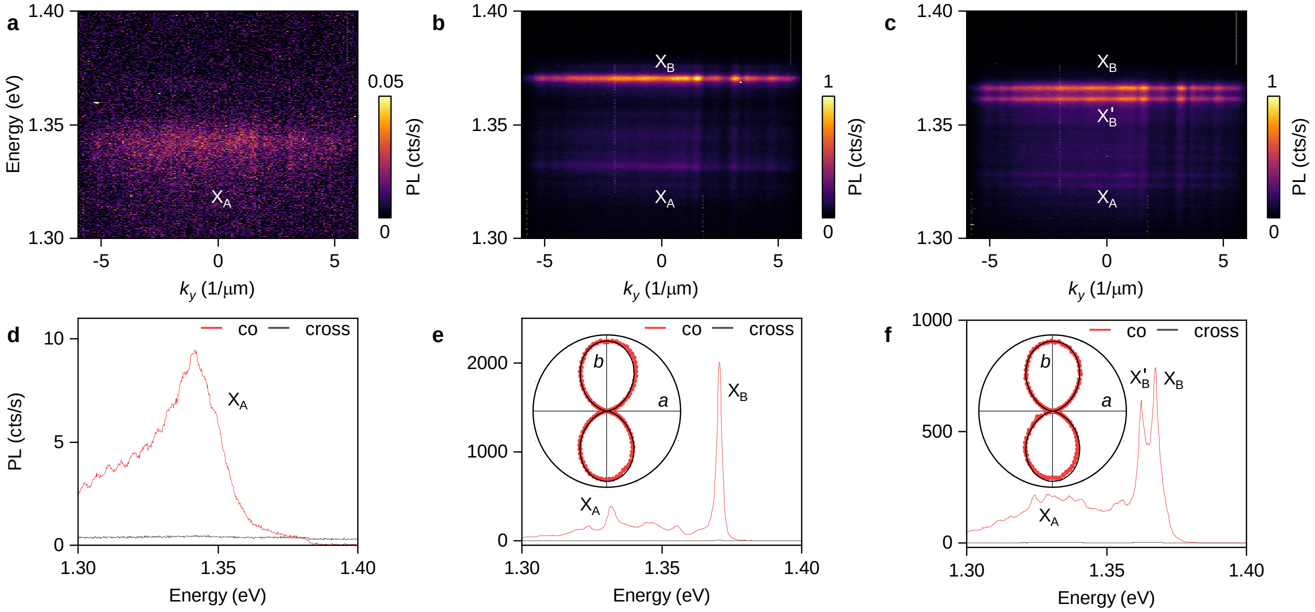

Supplementary Fig. 3. **Momentum-space and linear polarization data.** **a** – **c**, Momentum-space photoluminescence (PL) dispersions at 0 V of mono- (**a**), bi- (**b**), and trilayer (**c**). **d** – **f**, PL spectra of mono- (**d**), bi- (**e**), and trilayer (**f**) under linearly polarized excitation along the *b*-axis with co- and cross-polarized detection. Insets in **e** and **f** show the integrated PL intensity as a function of detection polarization angle together with a $\sin^2$ function.

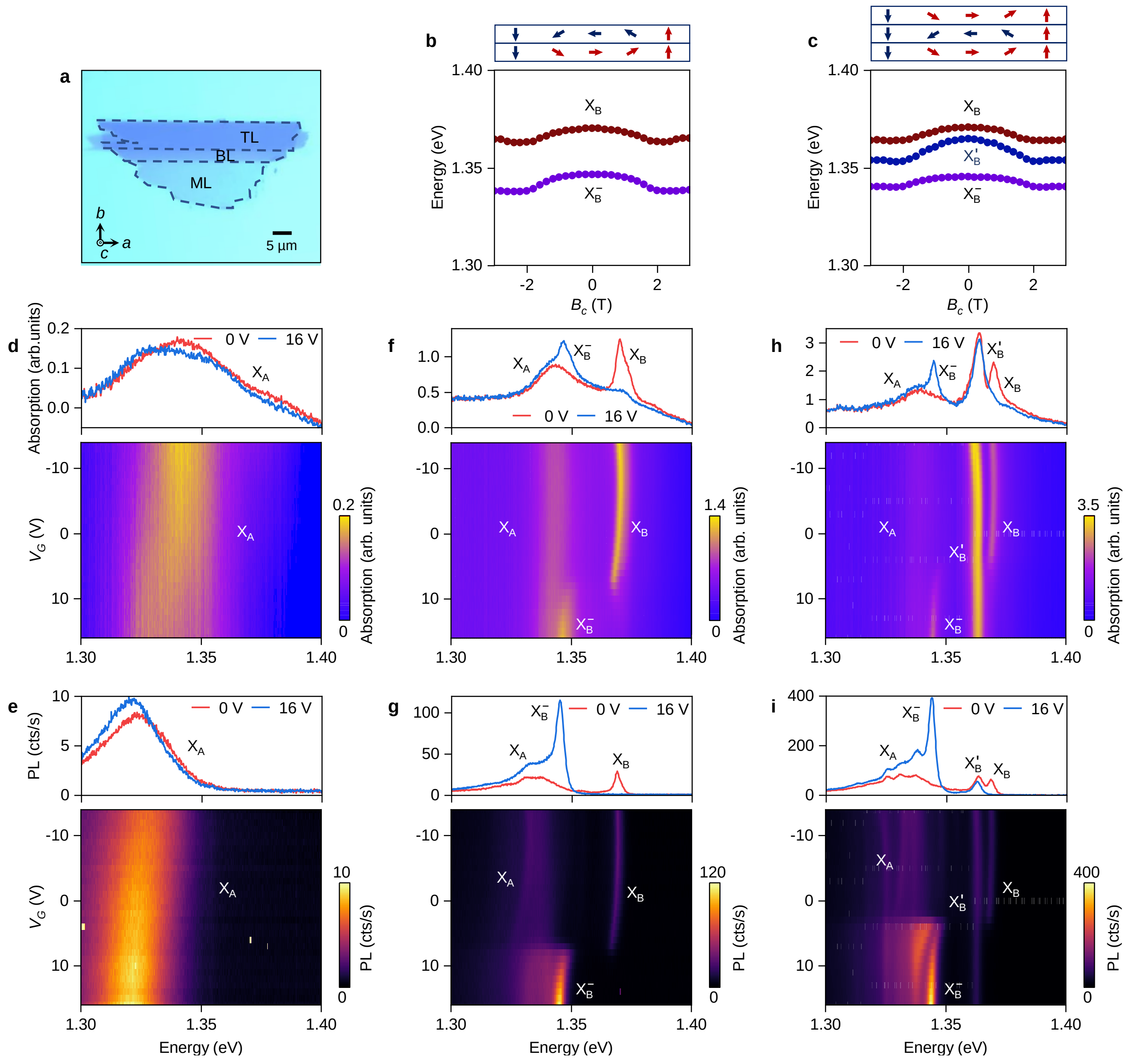

Supplementary Fig. 4. **Magneto-dispersions along the hard c-axis and doping-dependence for sample 2.** **a**, Optical micrograph of the CrSBr flake of sample 2, showing mono- (ML), bi- (BL), and trilayer (TL) regions, as well as the crystallographic axes. **b**, and **c**, Energies of X_B, X'_B, and X_B^- as a function of magnetic field along the c-axis (B_c) extracted from 0 and 16 V differential reflectance (DR) magneto-dispersions for bi-, and trilayer, respectively. Spin orientations are indicated in the top panels. **d**, and **e**, Monolayer absorption and photoluminescence (PL) spectra (top) and sweeps of the gate voltage V_G (bottom), respectively. **f** − **i**, Same as **d**, **e**, but for the bilayer (**f**, **g**) and trilayer (**h**, **i**). X_A, X_B, and X'_B label neutral and X_B^- charged exciton transitions. The absorption was determined by applying Kramers-Kronig relation to the differential reflectance. PL and DR for the monolayer were not measured at the same positions on the sample.

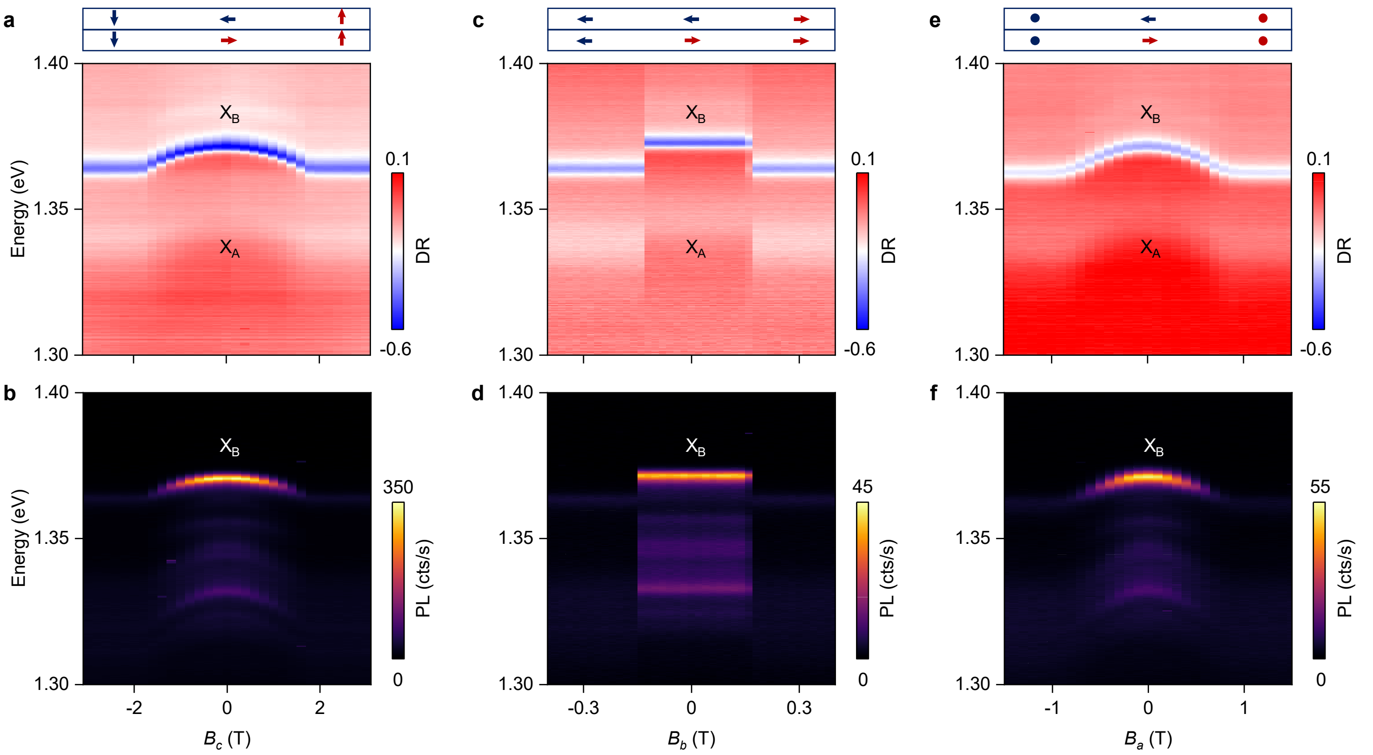

Supplementary Fig. 5. **Bilayer magnetic field dependence along** c**-,** b**-, and** a**-axes. a – f**, Differential reflectance (DR, **a**, **c**, **e**) and photoluminescence (PL, **b**, **d**, **f**) magneto-dispersions with magnetic fields along the hard c (**a**, **b**), easy b (**c**, **d**), and intermediate a axes (**e**, **f**) at 0 V. X_A, and X_B label neutral exciton transitions. Top panels in **a**, **c**, and **e** indicate the bilayer spin orientation, with blue and red arrows and dots representing layers with opposite spin orientation.

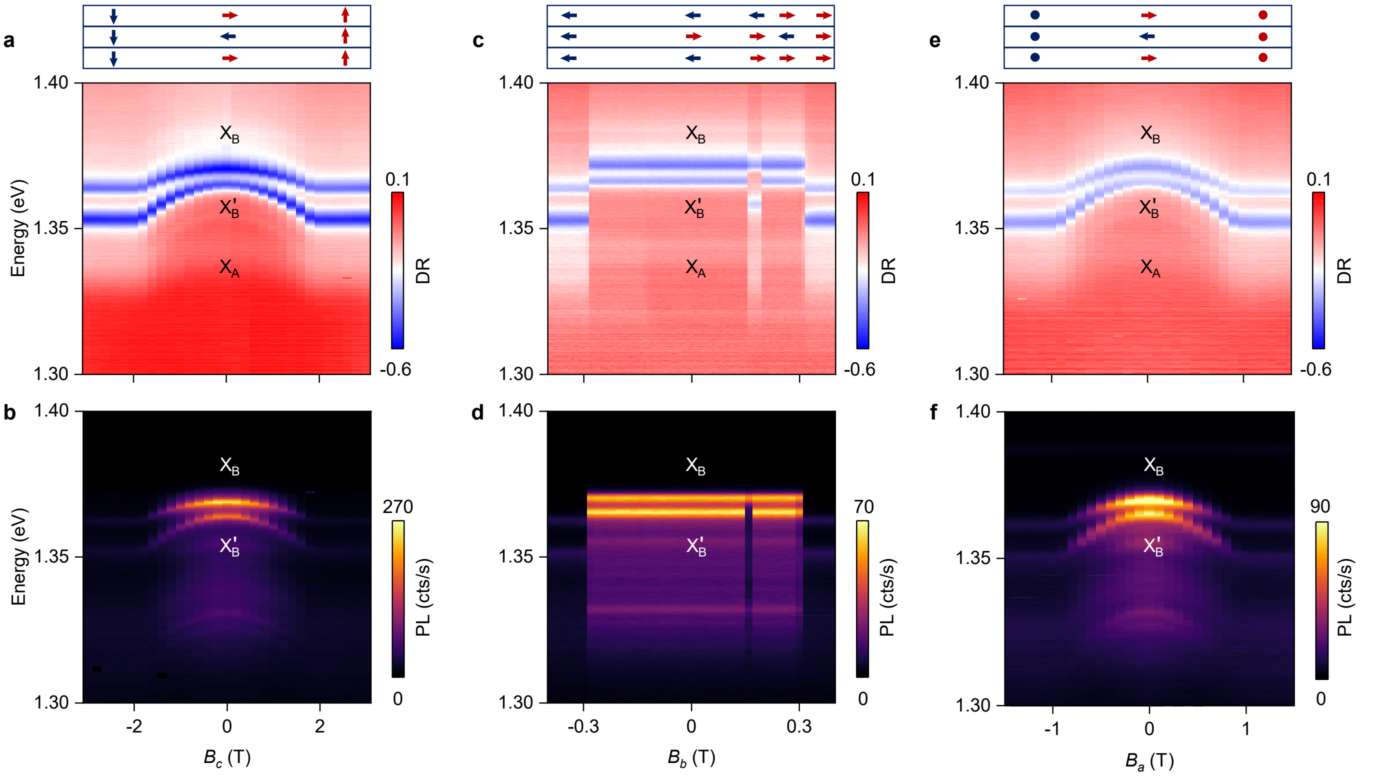

Supplementary Fig. 6. **Trilayer magnetic field dependence along** c**-,** b**-, and** a**-axes. a** – **f**, Differential reflectance (DR, **a**, **c**, **e**) and photoluminescence (PL, **b**, **d**, **f**) magneto-dispersions with magnetic fields along the hard c (**a**, **b**), easy b (**c**, **d**), and intermediate a axes (**e**, **f**) at 0 V. X_A, X_B, and X'_B label neutral exciton transitions. Top panels in **a**, **c**, and **e** indicate the trilayer spin orientation, with blue and red arrows and dots representing layers with opposite spin orientation.

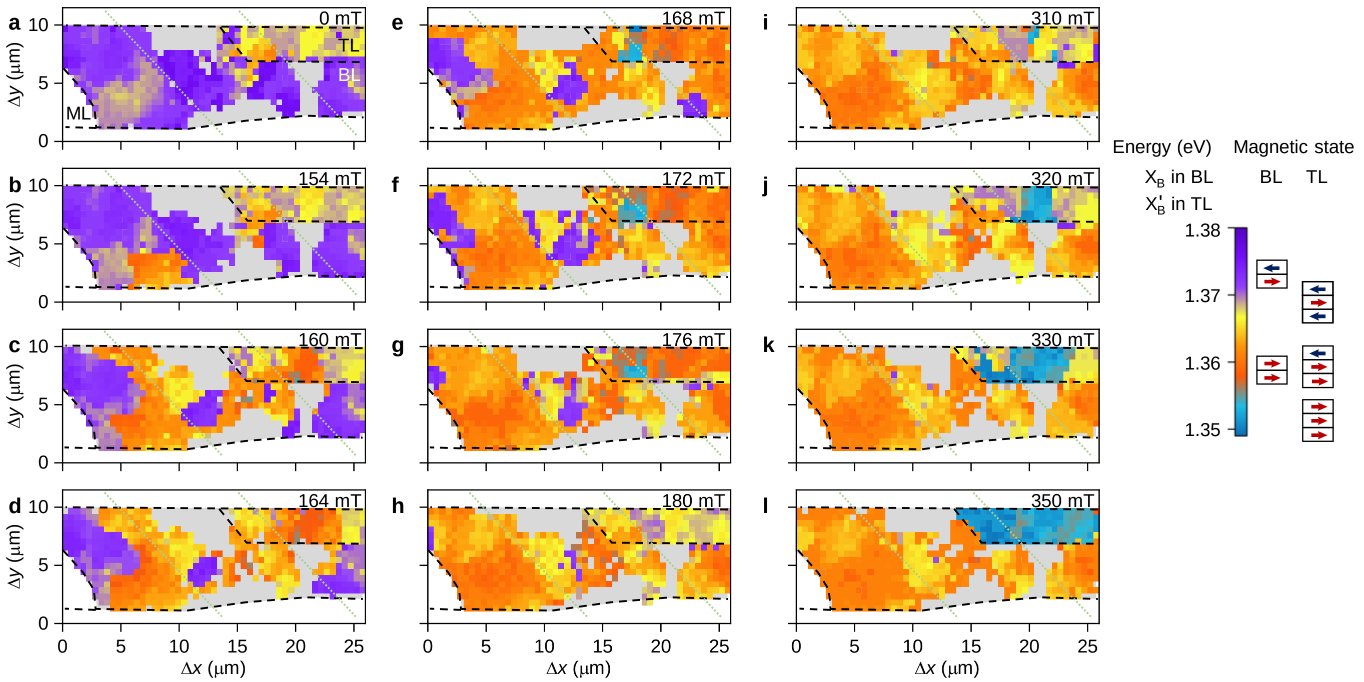

Supplementary Fig. 7. **Large-area magnetic domain imaging.** **a** – **l**, Spatial maps of a large area of the sample showing the energies of X_B and X'_B in bi- (BL) and trilayer (TL) regions, respectively, for different *b*-axis magnetic fields ranging from 0 mT (**a**) to 350 mT (**l**), extracted from hyperspectral differential reflectance maps. The color bar indicates the energies of the different spin orientations, with blue and red arrows representing layers with left and right spin orientation, respectively. Outlines of mono-, bi- and trilayer are marked with black dashed lines and the few-layer graphene contact is indicated by green dotted lines. White areas indicate regions away from bi- and trilayer. Grey areas signal the absence of excitonic resonances.
